# Enhanced High Performance of a Metasurface Polarizer Through Numerical Analysis of the Degradation Characteristics

**DOI:** 10.1186/s11671-018-2627-x

**Published:** 2018-07-31

**Authors:** Hiroyuki Kurosawa, Bongseok Choi, Masanobu Iwanaga

**Affiliations:** 1 National Institute for Materials Science (NIMS), 1-1 Namiki, Tsukuba, 305-0044 Japan; 2 National Institute of Information and Communications Technology (NICT), 588-2 Iwaoka, Kobe, 651-2492 Japan; 3 Present address: Materials and Devices Advanced Research Institute, LG Electronics, Seoul, South Korea

**Keywords:** Metasurface, Polarizer, Plasmonics

## Abstract

This study focuses on the experimental and numerical investigations for the degradation characteristics of a metasurface polarizer. The metasurface has a stacked complementary structure that exhibits a high extinction ratio of the order of 10,000 in the near-infrared region. However, its performance has significantly degraded over time. To clarify the origin of this degradation, the effects of surface roughness and metallic loss are investigated numerically. The degradation is mainly attributed to increase in the loss. These numerical calculations also reveal that the extinction ratio is enhanced by adjusting the thicknesses of the complementary structures to different values. This study paves a way to realize a metasurface polarizer that has a low sensitivity to the time degradation and has a high extinction ratio.

## Background

The control of light on the nanoscale has been investigated widely in nano-optics and nanophotonics. As a result, different types of photonic nanostructures have been proposed so far. For instance, the photonic crystal (PhC) nano-cavities with ultra-high quality (Q) factors [1] can confine light into a subwavelength region. Similar to the PhC cavities, high Q factors are realized by micro-disk [2, 3], spherical [4], and troidal [5] cavities. Those cavities with high Q factors usually consist of transparent dielectric materials. In contrast to those dielectric cavities, metallic cavities have low Q factors but can reduce their entire cavity sizes. In particular, plasmonic subwavelength cavities are important for controlling light on an extremely small scale [6]. Although plasmonic cavities have low Q factors, they can squeeze light into a deep subwavelength region [7]. This extremely confined light is anticipated to be a key to merge photonics and electronics [8].

In addition to the abovementioned photonic nanostructures, metasurfaces have been recently attracting a considerable attention for designing highly functional and ultra-thin optical devices. There are various types of metasurfaces that control refraction [9], reflection [10], photoluminescence [11], fluorescence [12–14], waveplates [15], and beam splitters [16]. Polarization state is one of the fundamental and important properties of light that can be controlled by metasurfaces [17–22]. Numerical and experimental studies have shown that a metasurface polarizer with a stacked complementary structure has a high extinction ratio of the order of 10,000 in the near-infrared region [23–26]. The complementary structures have resonances at nearly the same wavelength due to Babinet’s principle [27, 28]. When a complementary structure is on resonance that exhibits a high transmittance for a specific polarization, the other structure is off resonance that exhibits a low reflectance for the same polarization. As a result, the whole structure exhibits a high transmittance. For the orthogonal polarization, the role of the electric and magnetic fields exchanges, resulting in the high reflectance. Thus, the metasurface with complementary structures exhibits a high extinction ratio. However, there is a deep concern about the stability and reliability of this high performance because the metasurface comprises silver, which degrades in the atmosphere. To circumvent this problem, an alternative approach is to use gold as a plasmonic material but this diminishes the performance of the polarizer due to increased metallic loss. Therefore, for practical applications, the stability and reliability of the metasurface polarizer should be addressed.

In this study, we investigate the degradation characteristics of the metasurface polarizer. We show that the extinction ratio of the polarizer exhibits time degradation. As an origin of the degradation, we focus on the effect of surface morphology on the high performance of the polarizer. To describe the morphology, we introduce two models. One describes surface roughness by a periodic curve with a Gaussian white noise, while the other describes the roughness by using randomly distributed nanoparticles. We also investigated the effect of metallic loss on the high performance. Throughout these numerical calculations, we reveal a crucial factor that causes the degradation and propose an optimized metasurface polarizer with a high extinction ratio.

## Methods/Experimental

**Fig. 1:**
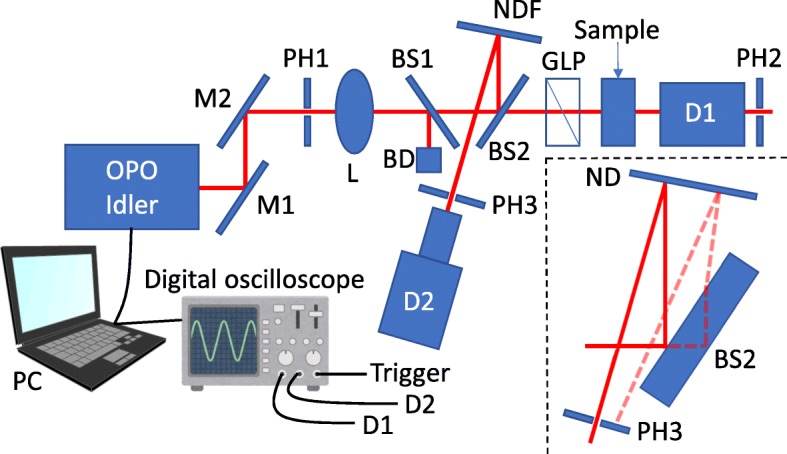
A schematic of the experimental setup of the extinction ratio measurement. M mirror, PH pinhole, L lens, BS beam sampler, BD beam damper, NDF neutral density filter, GLP Glan-laser prism, D detector

The experimental setup for the high extinction ratio measurement is schematically shown in Fig. 1. We used an optical parametric oscillator (OPO) pumped by a frequency-tripled Nd:YAG (yttrium iron garnet) laser (Optolette 355, Opotek) as a light source. The pulse width and repetition rate were 7 ns and 20 Hz, respectively. The idler light from the OPO was focused on the sample by a lens and was linearly polarized by a Glan-laser prism (GLP). The transmitted idler light was measured by extended InGaAs photodetector (Edmund Optics). In this optical system, the fluctuation of light intensity from a single pulse causes a poor signal-to-noise (S/N) ratio. Therefore, to remove the effect of this fluctuation, we measured the mean transmittance of a single pulse. To monitor the light intensity of a single pulse, we inserted a pair of beam samplers between the lens and the GLP. A portion of the idler light was reflected at the second beam sampler (BS2) and then reflected again at the reflective neutral density (ND) filter, which adjusted the reflected laser intensity not to damage a photodetector. The tuned laser was incident on an extended InGaAs photodetector (Edmund Optics) through a pinhole, which blocked unnecessary “ghost” light reflected at the back surface of the BS2 (see the inset of Fig. 1). The first beam sampler serves as a compensator of deviation of the optical path.

Using this setup, we evaluated the extinction ratio as follows. The transmitted signal is calculated to be *D*_1_=(1−*R*_BS2_)*T*_GLP_*T*_Sample_*I*, where *R*_BS2_, *T*_GLP_, *T*_Sample_, and *I* are the reflectance of the BS2, transmittance of the GLP, transmittance of the sample, and the light intensity in front of the BS2, respectively. The signal intensity of the detector 2 is calculated to be *D*_2_=*R*_BS2_*R*_NDF_*I*, where *R*_NDF_ is the reflectance of the reflective ND filter. Note that the light intensity is decreased sufficiently so that the detected signal is proportional to the light intensity. Using *D*_1_ and *D*_2_, we can calculate *T*_Sample_ as

1 $$\begin{array}{@{}rcl@{}} T_{\text{Sample}} = \frac{R_{\mathrm{BS2}}R_{\text{NDF}}}{1-R_{\mathrm{BS2}}}\frac{1}{T_{\text{GLP}}}\frac{D_{1}}{D_{2}}. \end{array} $$

To evaluate *T*_Sample_, we also need to measure the reflectance and transmittance of the optical elements, such as the beam sampler. This is unnecessary because our focus is on an extinction ratio, namely the ratio of transmittance. By rotating the sample 90° and measuring the transmittance by the same setup, we can simply obtain the extinction ratio *η* as

2 $$\begin{array}{@{}rcl@{}} \eta = \frac{T_{\text{Sample}}^{\mathrm{H}}}{T_{\text{Sample}}^{\mathrm{L}}} = \frac{(D_{1}/D_{2})^{\mathrm{H}}}{(D_{1}/D_{2})^{\mathrm{L}}}, \end{array} $$

where the superscripts H and L indicate the polarization states that exhibit high and low transmittance, respectively. In this paper, we measured the ratio *D*_1_/*D*_2_ for the orthogonal polarization states and evaluated the extinction ratio *η*.

To confirm the validity of the measured data, we performed numerical calculations based on the rigorous coupled-wave analysis (RCWA) incorporated with the scattering matrix method [29, 30] and an inverse Fourier method [31]. The permittivities of Ag and silica were obtained from [32] and [33], respectively. The number of reciprocal lattice vectors used in the calculation was 2601.

To calculate transmittance of rough metallic structures, we used a commercial software of COMSOL Multiphysics, which is based on the finite element method. In the previous study [34], the effects of the roughness on the optical response are described by the increase in the imaginary part of the permittivity of metal. In this paper, in addition to the increase in the metallic loss, we also considered the direct effects of structural changes followed by the roughness on the transmittance. We dealt with these two effects separately. When considering only the effects of the structural changes, we applied bulk permittivity to metallic structures with roughness. On the other hand, when considering only the effects of the increased loss, we applied the modified permittivity to metallic structures without roughness. We set the relative tolerance of the numerical calculations to be less than 1%.

## Results and Discussion

**Fig. 2:**
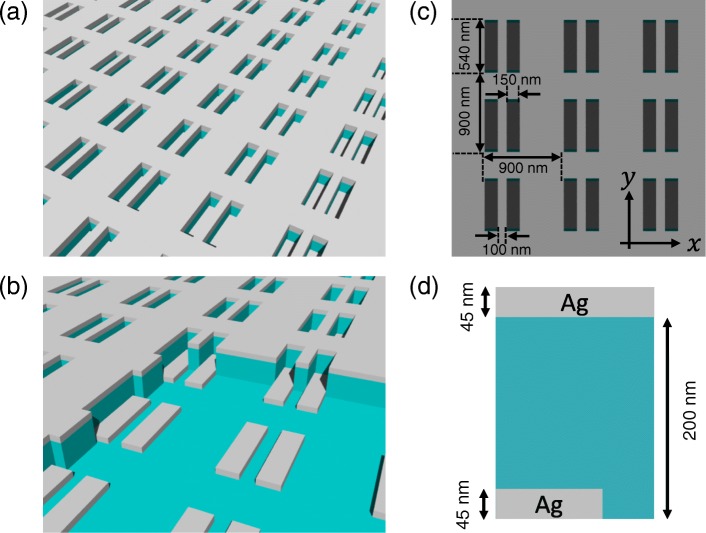
A schematic of the metasurface polarizer (**a**) that comprises three layers (**b**). The metasurface has an array of rectangular hole pair with a period of 900 nm in the *x* and *y* directions (**c**). The thicknesses of the metallic and dielectric layers are 45 and 200 nm, respectively (**d**)

**Fig. 3:**
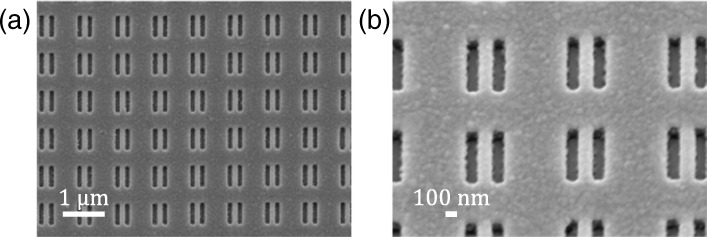
The SEM image of **a** the metasurface polarizer and **b** its magnified image

Figure 2a depicts the schematic of the three-layered metasurface polarizer. The first layer has a complementary structure to the third layer (see Fig. 2b), with both layers comprising silver (Ag). The second layer and the substrate comprise silica (SiO_2_). As shown in Fig. 1c, the metasurface has an array of a rectangular hole (150 nm × 540 nm) pair and has a period of 900 nm in the *x* and *y* directions. The thicknesses of the metallic and dielectric layers are 45 and 200 nm, respectively (see Fig. 2d). The sample was prepared by nanoimprint lithography coupled with subsequent dry etching techniques [35]. The details of the sample preparation are described in [26]. Figure 3 shows the scanning electron microscope (SEM) images of the prepared sample.

**Fig. 4:**
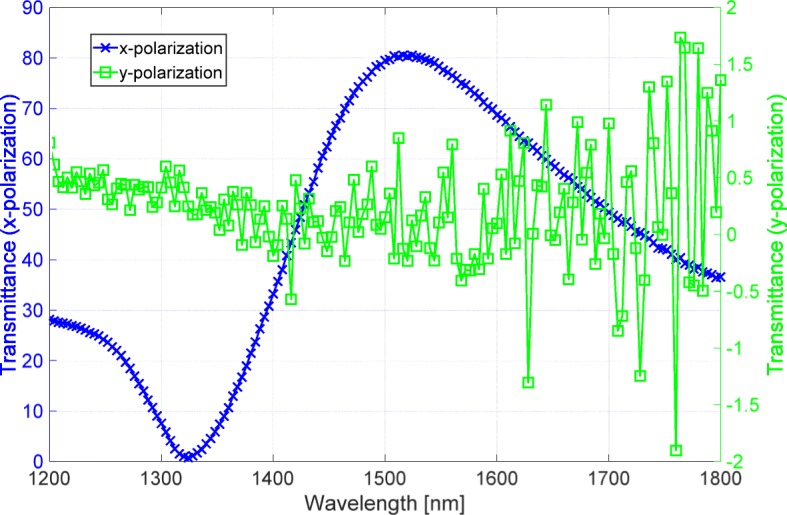
Transmittance spectra measured by the spectrophotometer. The blue and green lines are the spectra for the *x* and *y* polarizations, respectively

We used a spectrophotometer (V-7200, JASCO, Japan) to measure the transmittance of the sample for the *x* and *y* polarizations. Figure 4 shows the measured results. The blue and green lines indicate the transmittance for the *x* and *y* polarizations, respectively. The blue line that corresponds to the high transmittance is measured with a high S/N ratio. However, the green line corresponding to the low transmittance suffers from a low S/N ratio, thus indicating that the polarizer has a high extinction ratio. In particular, the green line has negative signals at wavelengths longer than 1350 nm because the intensity of transmitted light is under the spectrophotometer’s noise level. Hence, we used the optical system described in the previous section to measure the high extinction ratio.

**Fig. 5:**
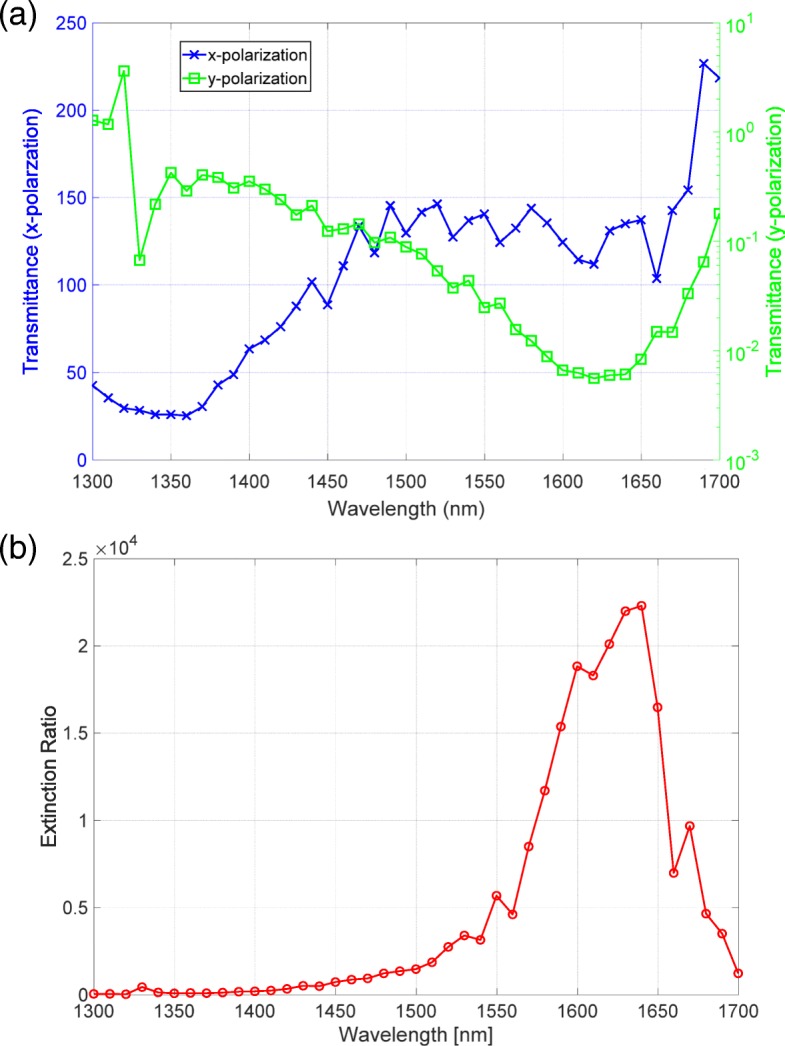
**a** Transmittance spectra for the *x* (blue) and *y* (green) polarizations measured by the setup shown in Fig. 1. **b** The extinction ratio spectrum of the metasurface polarizer

Figure 5a shows the measured transmittance spectra for the *x* and *y* polarizations. The blue line that corresponds to the high transmittance has a similar spectral profile as the transmittance measured by the spectrophotometer. The green line corresponding to the low transmittance has a clear dip around the wavelength of 1625 nm, which was not measured by the spectrophotometer. By dividing the transmittance for the *x* polarization by that for the *y* polarization, we evaluated the extinction ratio spectrum shown in Fig. 5b. The extinction ratio spectrum has a peak value exceeding 20,000 around the wavelength of 1640 nm.

**Fig. 6:**
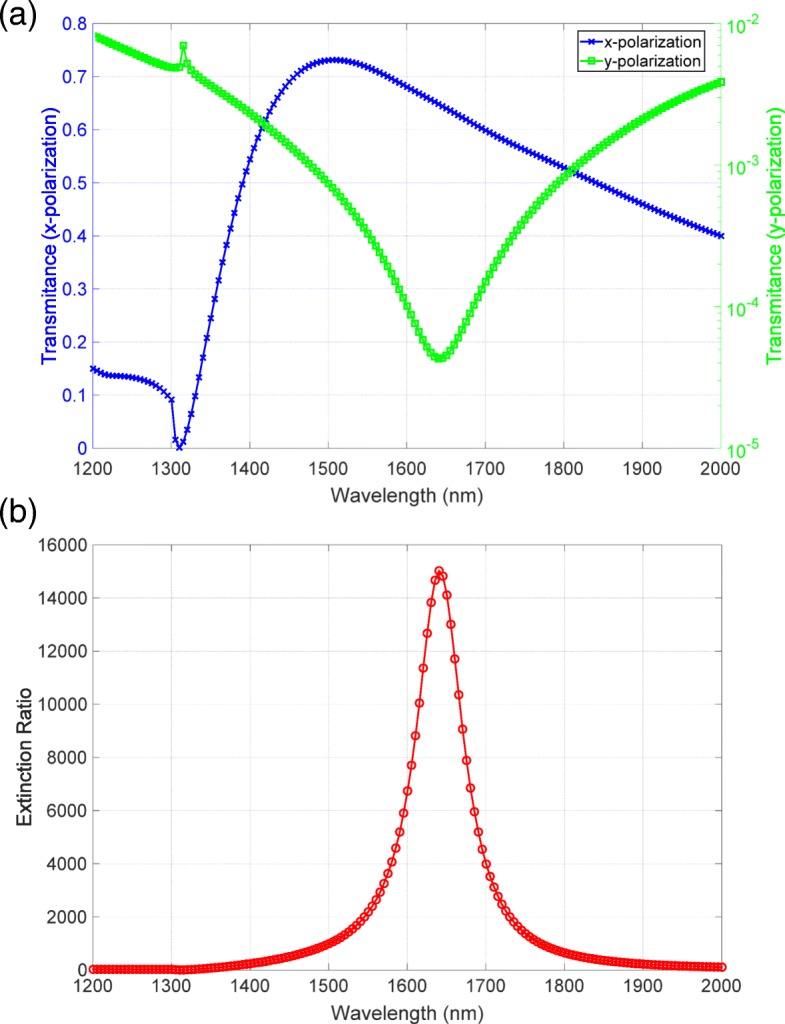
Numerical calculation results of **a** transmittance and **b** extinction ratio spectra. The blue and green lines in **a** correspond to the *x* and *y* polarizations, respectively

To consider the validity of the measured data, we compared the measured spectra to the numerical calculation results. As shown in Fig. 6a, the high transmittance spectrum was consistent with the spectra measured by the spectrophotometer. The low transmittance spectrum, which is displayed in a logarithmic scale, has a clear dip around the wavelength of 1640 nm. This feature agreed well with that in the observed spectrum. The extinction ratio spectrum shown in Fig. 6b has a peak of 15,000, which is close to the observed value. Thus, the measured transmittance and extinction ratio spectra were consistent with the results of numerical calculation, indicating that we had successfully observed the high extinction ratio exceeding 20,000.

**Fig. 7:**
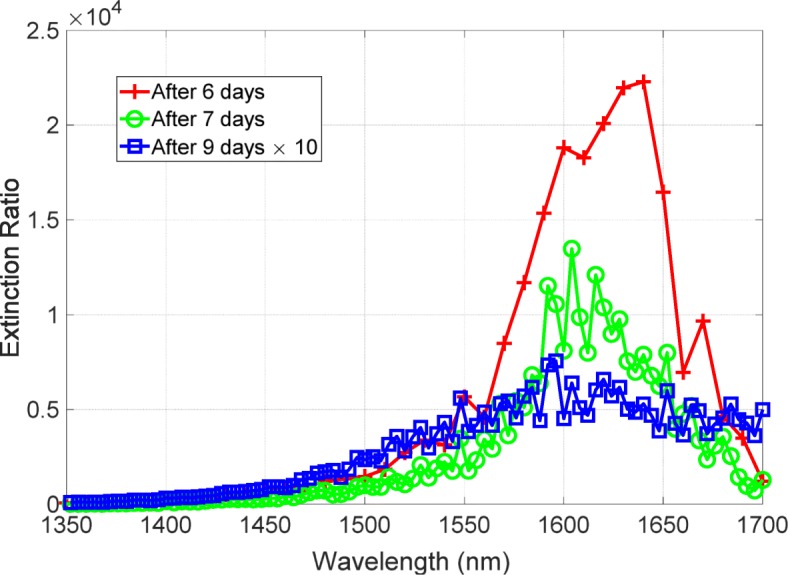
Time degradation of the extinction ratio. The red, green, and blue lines are the extinction ratio spectra for 6, 7, and 9 days after the metal deposition

Following the experimental demonstration of the metasurface with the high extinction ratio, we focus on the the stability to time degradation because the metasurface comprise Ag, which is subject to degradation in the atmosphere. Figure 7 shows the time degradation of the extinction ratio. The red, green, and blue lines are the extinction ratio spectra observed after 6, 7, and 9 days after metal deposition, respectively. The red line has a peak value exceeding 20,000. After a single day of the red line measurement, the extinction ratio degraded but still had a peak value exceeding 10,000. However, two days after the measurement of the green line, the extinction ratio significantly degraded and had a peak value of 500. The blue line has a broadened line width, indicating that an increase in loss would be involved in this degradation. Thus, the extinction ratio exhibited a drastic degradation and the performance degraded one order of magnitude. We also found the blue-shifted peaks of the extinction ratio spectra following the degradation. A study of crucial factor involving degrading the performance is described.

The degradation proceeded rapidly, and the line widths of the extinction spectra broadened, indicating that some structural changes would be involved in this degradation process. Therefore, we investigate the manner in which the surface morphology of the metallic nanostructure affects the performance of the polarizer. To describe the morphology, we introduce two models. One describes the surface by a periodic curve with a Gaussian white noise and the other by randomly distributed nanoparticles.

**Fig. 8:**
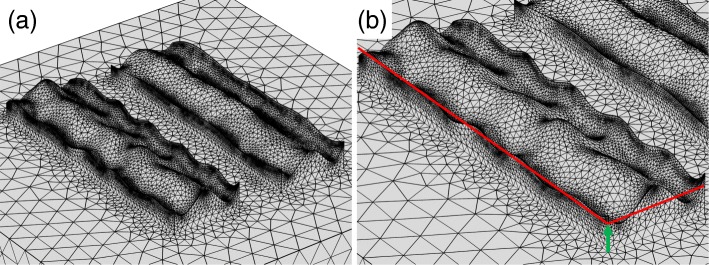
**a** Roughed surface modeled using a periodic curve with a Gaussian white noise. **b** The base thickness indicated by the green arrow is varied in the calculation

**Fig. 9:**
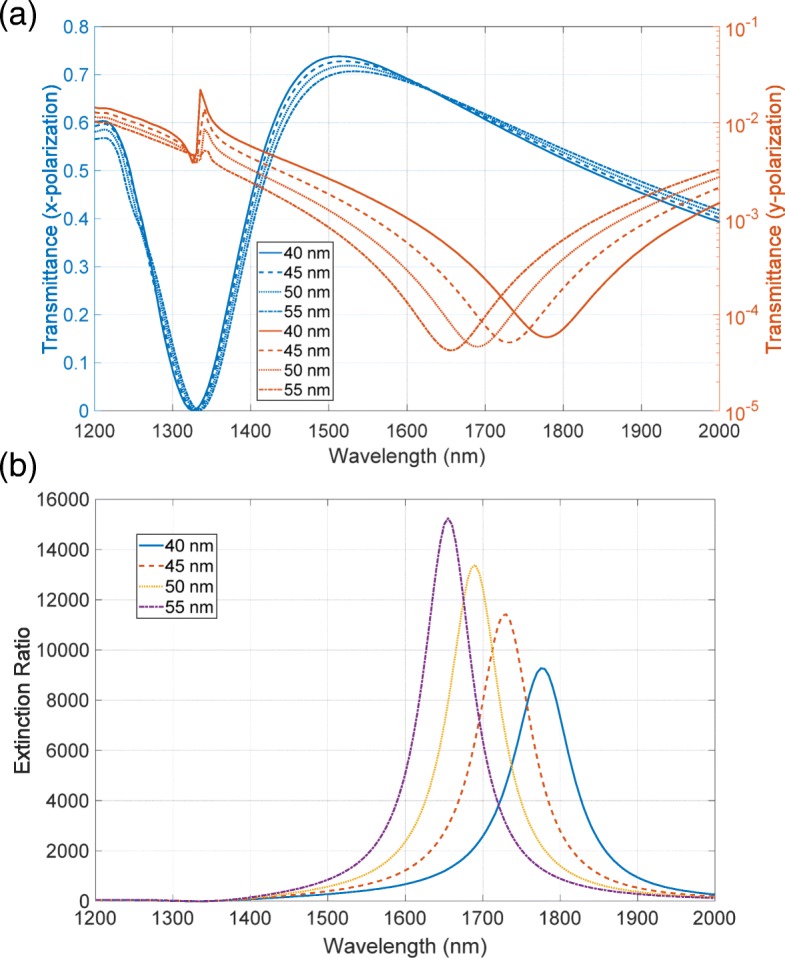
**a** Transmittance and **b** extinction ratio spectra of the first model shown in Fig. 8. The base thickness of the bottom metallic layer is varied from 40 to 55 nm with a step of 5 nm

First, we investigate the model using the periodic curve. Figure 8a depicts the modeled surface. We introduced the roughness only in the bottom metallic layer to save CPU time and memory resources. Due to the rough surface, the effective thickness of the metallic layer varies. Hence, we varied the thickness of the bottom layer indicated by the green arrow in Fig. 8b. Figure 9a, b shows the transmittance and extinction ratio spectra of this structure, respectively. Even in the presence of the roughness, the metasurface polarizer has high extinction ratios of the order of 10,000, indicating that roughness does not significantly degrade the performance. The numerical calculations have also shown the red-shifted spectra of the extinction ratio with the decrease in the thickness. This red-shift is elucidated by the spectral features of transmittance shown in Fig. 9a. The high transmittance has greatly low sensitivity in relation to the variation in the metal thickness, while the low transmittance has the red-shifted dip position with the decrease in the thickness. The peak position of the extinction ratio depends on the dip of the low transmittance, resulting in the red-shift. The red-shift that appeared in the calculation does not agree with the experimentally observed feature of the blue-shift.

**Fig. 10:**
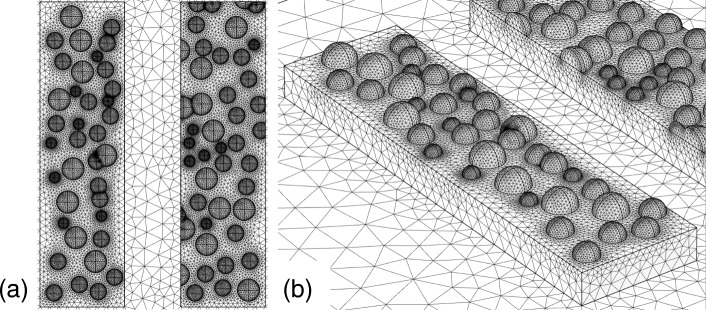
**a** The top view and **b** bird’s eye view of the rough surface modeled using randomly distributed nanoparticles

**Fig. 11:**
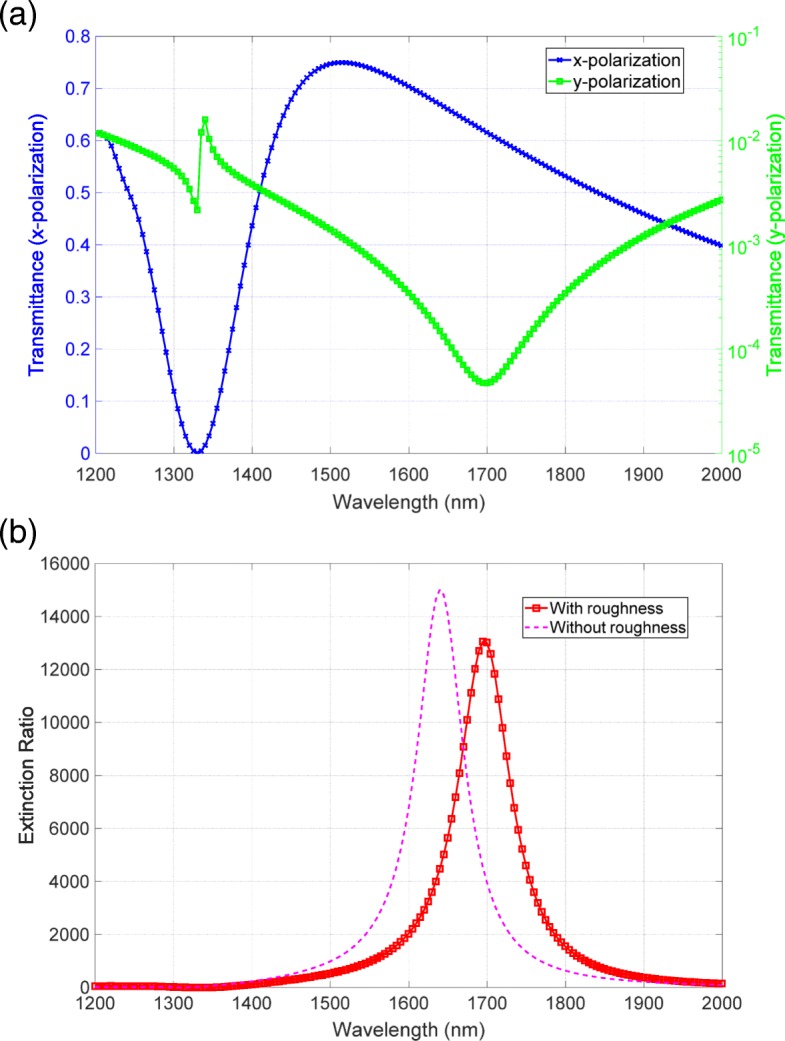
**a** Transmittance and **b** extinction spectra of the second model shown in Fig. 10

Second, we investigate the model by the nanoparticles. Figure 10a depicts the modeled surface, where nanoparticles with radii of 15, 20, and 25 nm are distributed randomly on the surface of the bottom metallic structure, as shown in Fig. 10b. We placed the hemisphere-shaped nanoparticles on the surface in accordance with uniformly distributed random numbers. Under the random distribution, some of the particles have slight spatial overlap and the mesh size between the particles becomes extremely memory-consuming. In this case, to save the memory, we manually shifted one of the particles and lowered the mesh size. We set the thickness of the bottom structure to be 40 nm. Figure 11a, b shows the transmittance and extinction ratio spectra of this structure, respectively. Similar to the first model, the extinction ratio spectrum has a peak value of the order of 10,000, and does not significantly degrade. The red-shifted peak has also appeared in the presence of the nanoparticles. These features are also the same as those observed in the first model, but they do not agree with the experimental result of the degradation characteristics and the blue-shift.

**Fig. 12:**
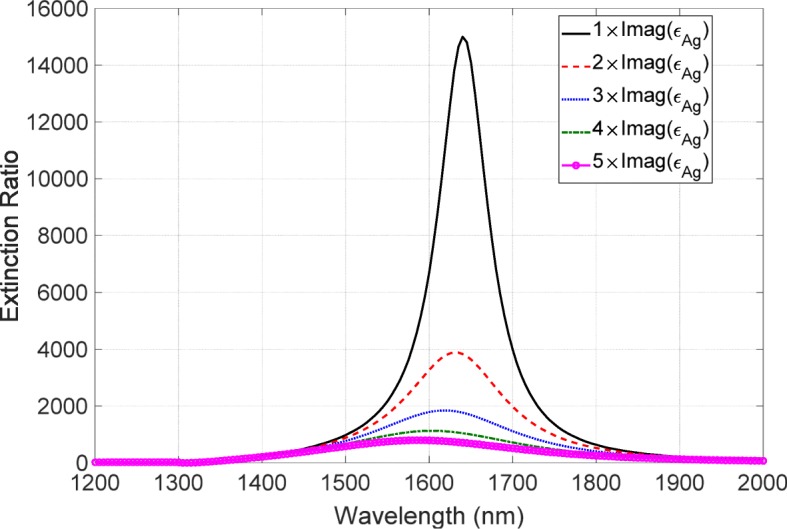
Metallic loss dependence of the extinction spectra. The black, red, blue, green, and magenta lines correspond to the cases of *C*=1,2,3,4, and 5, respectively

At this stage, we have numerically shown that the variation in surface morphology does not significantly degrade the performance of the metasurface polarizer. This robustness of the morphology is attributed to Babinet’s principle. Babinet’s principle does not refer to the surface morphology but it refers to the screens of the complementary structures. The high-performance polarizer based on this principle is not strongly affected by the morphology because the screens are invariant even in the presence of the surface roughness, resulting in the robustness to the morphology. Therefore, as an origin of the degradation, we need to consider another effect of the surface morphology. Here, we focus on the metallic loss related to the morphology. With the increase in the surface roughness, the imaginary part of Ag increases due to the surface scattering and grain boundary effects [36, 37]. This increase in the loss is explained by the damping constant of Drude model described as *γ*=*ρne*^2^/*m*_e_, where *ρ*, *n*, *e*, and *m*_e_ are respectively the electrical resistivity, electron density, electron charge, and effective electron mass. The resistivity consists of two terms. One is bulk resistivity and the other is surface one. The surface resistivity *ρ*_s_ is inversely proportional to the lateral correlational length *ξ*, namely, *ρ*_s_∝*ξ*^−1^ [38]. With the increase in roughness, the lateral correlation length *ξ* decreases, thus resulting in the higher surface resistivity and metallic loss. This physical mechanism was not included in the calculation because a periodic boundary condition was used and a rough periodic structure was assumed. We consider the effects of this increase in metallic loss on the extinction ratio and modify the permittivity of Ag as follows:

3 $$\begin{array}{@{}rcl@{}} \tilde{\epsilon}_{\text{Ag}} = \text{Re}\left(\epsilon_{\text{Ag}} \right) + C\times \text{Im}\left(\epsilon_{\text{Ag}}\right)\mathrm{i}, \end{array} $$

where *ε*_Ag_ is the permittivity of Ag obtained from [32], *C* is a constant representing the increase in the metallic loss, and i denotes an imaginary unit. Note that the real part of the permittivity must be modified following the increase in the imaginary part because the real and imaginary parts are connected by the Kramers–Kronig relationship. In this study, we modified only the imaginary part to have a qualitative discussion. Using this modified permittivity, we calculate the extinction ratio spectrum. This result is shown in Fig. 12, in which the constant *C* is varied from 1 to 5. The extinction ratio drastically decreases with an increase in the metallic loss. In addition, the peak position of the spectrum exhibited the blue-shifted feature with the increase in the loss. These features of the drastic degradation and the blue-shift agree well with the experimentally observed features. The origin of this blue-shift is elucidated as follows. The dip value of the low transmittance becomes increasingly shallow with the increase in the metallic loss. As a result, the contribution from the peak value of the high transmittance to the extinction ratio increases. The peak position has a strong insensitivity to the metallic loss and is at the shorter wavelength than the dip position, resulting in the blue-shift of the extinction spectrum. Thus, we have found that the increase in the imaginary part is a crucial factor responsible for the degradation.

**Fig. 13:**
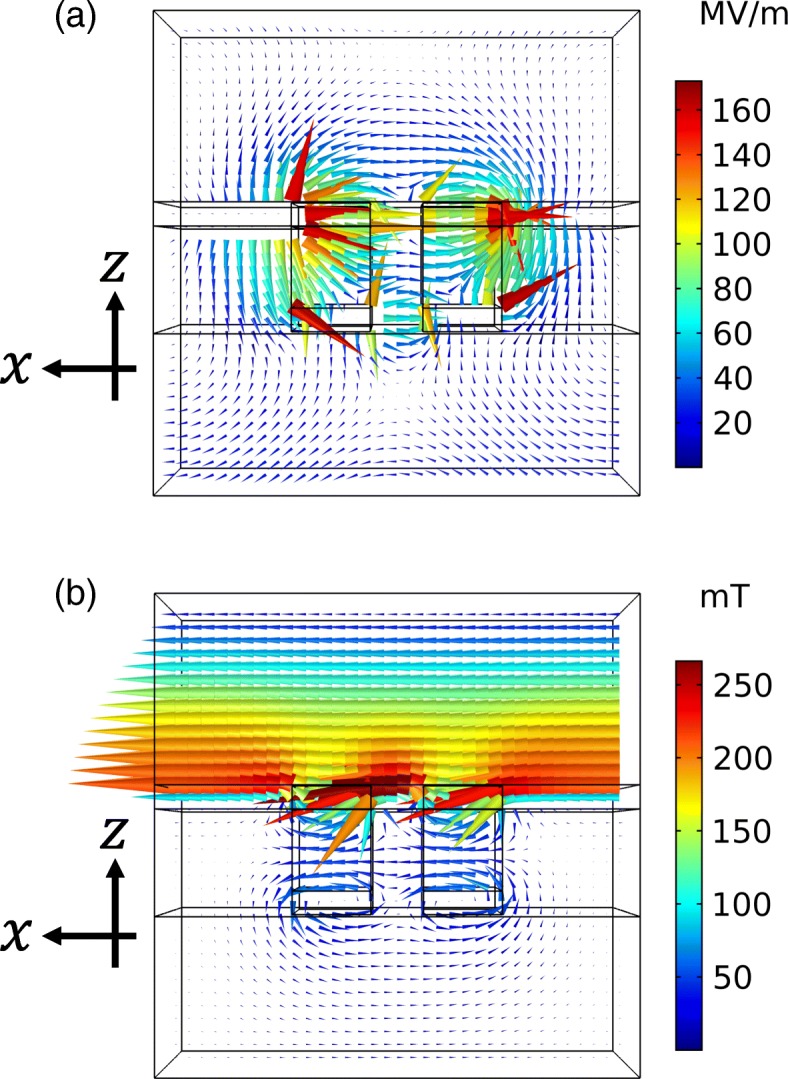
**a** Electric field distribution pattern at the peak of the high transmittance shown in Fig. 6. **b** Magnetic field distribution pattern at the dip of the low transmittance shown in Fig. 6. The pseudo color indicates the intensity of the vector field

**Fig. 14:**
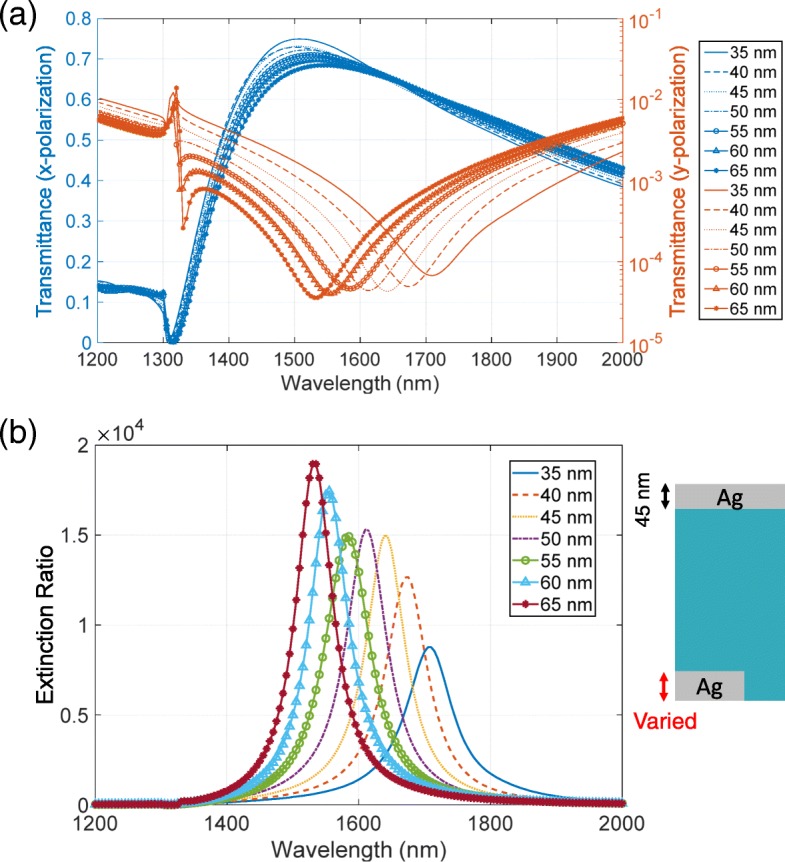
**a** Transmittance and **b** extinction ratio spectra when the complementary metallic layers have different thicknesses. The thickness of the top metallic layer is fixed to be 45 nm, while that of the bottom layer is varied from 35 to 65 nm with a step of 5 nm (see the inset in **b**)

We propose that the extinction ratio is enhanced by varying the thicknesses of the complementary metallic layers. The peak position of the high transmittance is located at a shorter wavelength than the dip position of the low transmittance. To enhance the extinction ratio, these peak and dip positions should be close to each other. According to Babinet’s principle, the peak and dip must be at the same wavelength. However, the principle assumes that complementary structures comprise a perfect electric conductor with an infinitely thin thickness, which is difficult to validate in the optical region even under approximation. As a result, the complementary structures have different resonance wavelengths. To adjust the wavelengths, we consider the characteristics of the eigenmodes responsible for the resonances. Figure 13a, b shows the electric and magnetic field distribution patterns at the peak and dip positions of the transmittance shown in Fig. 6, respectively. These field distributions are depicted in the *z*−*x* plane at *y*=0 under the incident light intensity of 1 W. The eigenmode of the high transmittance has a characteristic of an electric dipole in the top metallic structure, while that of the low transmittance has a characteristic of magnetic loop in the bottom structure. The resonance wavelength for the high transmittance is determined by the width of the air hole in the *z*−*x* plane. This is a fixed parameter and impossible to adjust. On the other hand, the resonance wavelength for the low transmittance is determined by the cross section of the bottom structure in the *z*−*x* plane. This is adjustable by varying the metal thickness. These adjustments are consistent with the thickness dependence of the transmittance that the peak position of the high transmittance has a low sensitivity to the thickness of the bottom metallic layer while the dip position of the low transmittance has a high sensitivity. Based on this analysis, we adjust the wavelength as follows. With the increase in the thickness, the cross section increases and the resonance wavelength of the low transmittance shifts to shorter wavelengths. As a result, the peak and dip positions become close and the extinction ratio is enhanced. To confirm this, we calculate the dependence of transmittance and extinction ratio spectra on the thickness. In this calculation, we fixed the thickness of the top metallic layer to be 45 nm. Figure 14a shows the transmittance spectra for the *x* and *y* polarizations. With the increase in the thickness, the dip position of the low transmittance shifts to shorter wavelengths and the dip becomes deeper. On the other hand, the peak position of the high transmittance is not strongly affected by varying the thickness even though the peak value decreases by ∼ 5%. Figure 14b shows the extinction ratio spectra. When the thickness is 35 or 40 nm, the dip of the low transmittance becomes shallower than that of 45 nm, resulting in the lower extinction ratio. When the thickness is 50 or 55 nm, there is almost no enhancement. This is because the enhancement by the adjustment of the peak and dip positions is canceled out by the decrease in the peak value of the high transmittance. When the thickness is 60 or 65 nm, there is a clear enhancement in the extinction ratio. This is due to the combination of the deeper dip value and the enhancement by the position adjustment. As we have numerically shown, further enhancement of the extinction ratio can be realized by adjusting the thicknesses of the complementary metallic structures. Such varying thicknesses could be realized by repeating metal deposition. First, metal deposition with a thickness of *a* is conducted on a patterned substrate. Then, by wiping with a clean cloth, only the top metallic layer is removed from the surface of the substrate with metal thickness of *a*. Subsequently, metal deposition with a thickness of *b* is conducted on the sample. As a result, the thicknesses of the top and bottom layers become *b* and *a*+*b*, respectively.

## Conclusions

We have investigated the degradation characteristics of the high-performance metasurface polarizer. The prepared metasurface exhibited a high extinction ratio of the order of 10,000. We noted that the high performance has degraded gradually. To clarify the origin of this degradation, we have investigated the effects of surface morphology on the extinction ratio. Two models were presented to describe the surface morphology. One models a rough surface by a combination of a periodic curve and a Gaussian white noise, while the other models the surface by randomly distributed nanoparticles. Both models indicated that the high performance did not degrade by the surface roughness. This is because the high extinction ratio is governed by Babinet’s principle, resulting in the robustness of the surface morphology. We have also investigated the relation between the extinction ratio and the increase in metallic loss because of the surface roughness, which showed drastic degradation of the extinction ratio. The spectral feature of the blue-shift was also reproduced by the numerical calculation, indicating that the degradation is due to the increase in the metallic loss. From this result, we find that the metal deposition should be conducted to reduce the scattering and grain boundary losses that are related to the roughness. Throughout the numerical calculation, we have found that the low transmittance has a high sensitivity to the surface morphology, while the high transmittance does not have the high sensitivity. By utilizing these findings, we proposed that the extinction ratio can be enhanced by varying the thicknesses of the metallic layers. This study paves a way for the development of a metasurface with a high performance and stability toward time degradation.

## Abbreviations

BS: Beam sampler
GLP: Glan-laser prism
NDF: Neural density filter
OPO: Optical parametric oscillator
PhC: Photonic crystal
SEM: Scanning electron microscope
YAG: Yttrium iron garnet

## References

[1] Akahane Y, Asano T, Song BS, Noda S (2005). Fine-tuned high-q photonic-crystal nanocavity. Opt Express.

[2] Soltani M, Yegnanarayanan S, Adibi A (2007). Ultra-high q planar silicon microdisk resonators for chip-scale silicon photonics. Opt Express.

[3] Kurosawa H, Kumano H, Suemune I (2015). Ultrahigh quality factor in a metal-embedded semiconductor microdisk cavity. Opt Lett.

[4] Vernooy DW, Ilchenko VS, Mabuchi H, Streed EW, Kimble HJ (1998). High-q measurements of fused-silica microspheres in the near infrared. Opt Lett.

[5] Armani DK, Kippenberg TJ, Spillane SM, Vahala KJ (2003). Ultra-high-q toroid microcavity on a chip. Nature.

[6] Yu Z, Gao Z, Song Z, Wang Z (2014). Terahertz spoof plasmonic coaxial microcavity. Appl Opt.

[7] Miyazaki HT, Kurokawa Y (2006). Squeezing visible light waves into a 3-nm-thick and 55-nm-long plasmon cavity. Phys Rev Lett.

[8] Ozbay E (2006). Plasmonics: merging photonics and electronics at nanoscale dimensions. Science.

[9] Yu N, Genevet P, Kats MA, Aieta F, Tetienne JP, Capasso F, Gaburro Z (2011). Light propagation with phase discontinuities: generalized laws of reflection and refraction. Science.

[10] Sun S, Yang KY, Wang CM, Juan TK, Chen WT, Liao CY, He Q, Xiao S, Kung WT, Guo GY, Zhou L, Tsai DP (2012). High-efficiency broadband anomalous reflection by gradient meta-surfaces. Nano Lett.

[11] Choi B, Iwanaga M, Sugimoto Y, Sakoda K, Miyazaki HT (2016). Selective plasmonic enhancement of electric- and magnetic-dipole radiations of er ions. Nano Lett.

[12] Iwanaga M, Choi B, Miyazaki HT, Sugimoto Y (2016). The artificial control of enhanced optical processes in fluorescent molecules on high-emittance metasurfaces. Nanoscale.

[13] Choi B, Iwanaga M, Miyazaki HT, Sugimoto Y, Ohtake A, Sakoda K (2015). Overcoming metal-induced fluorescence quenching on plasmo-photonic metasurfaces coated by a self-assembled monolayer. Chem Commun.

[14] Kurosawa H, Iwanaga M (2017). Optical-signal-enhancing metasurface platforms for fluorescent molecules at water-transparent near-infrared wavelengths. RSC Adv.

[15] Yu N, Aieta F, Genevet P, Kats MA, Gaburro Z, Capasso F (2012). A broadband, background-free quarter-wave plate based on plasmonic metasurfaces. Nano Lett.

[16] Guo Z, Zhu L, Guo K, Shen F, Yin Z (2017). High-order dielectric metasurfaces for high-efficiency polarization beam splitters and optical vortex generators. Nanoscale Res Lett.

[17] Iwanaga M (2012). Photonic metamaterials: a new class of materials for manipulating light waves. Sci Technol Adv Mater.

[18] Owiti EO, Yang H, Liu P, Ominde CF, Sun X (2018). Polarization converter with controllable birefringence based on hybrid all-dielectric-graphene metasurface. Nanoscale Res Lett.

[19] Wang BX, Zhao CY, Kan YH, Huang TC (2017). Design of metasurface polarizers based on two-dimensional cold atomic arrays. Opt Express.

[20] Song Z, Zhu J, Zhu C, Yu Z, Liu Q (2015). Broadband cross polarization converter with unity efficiency for terahertz waves based on anisotropic dielectric meta-reflectarrays. Mater Lett.

[21] Song Z, Zhang L, Liu QH (2016). High-efficiency broadband cross polarization converter for near-infrared light based on anisotropic plasmonic meta-surfaces. Plasmonics.

[22] Song Z, Chu Q, Shen X, Liu QH (2018). Wideband high-efficient linear polarization rotators. Front Phys.

[23] Iwanaga M (2010). Polarization-selective transmission in stacked two-dimensional complementary plasmonic crystal slabs. Appl Phys Lett.

[24] Iwanaga M (2010). Subwavelength electromagnetic dynamics in stacked complementary plasmonic crystal slabs. Opt Express.

[25] Iwanaga M (2010). Electromagnetic eigenmodes in a stacked complementary plasmonic crystal slab. Phys Rev B.

[26] Kurosawa H, Choi B, Sugimoto Y, Iwanaga M (2017). High-performance metasurface polarizers with extinction ratios exceeding 12000. Opt Express.

[27] Babinet M (1837). Compt Rend Acad Sci.

[28] Booker HG (1946). Slot aerials and their relation to complementary wire aerials (babinet’s principle). Electr Eng IIIA Radiolocation J Inst.

[29] Tikhodeev SG, Yablonskii AL, Muljarov EA, Gippius NA, Ishihara T (2002). Quasiguided modes and optical properties of photonic crystal slabs. Phys Rev B.

[30] Li L (1996). Formulation and comparison of two recursive matrix algorithms for modeling layered diffraction gratings. J Opt Soc Am A.

[31] Li L (1997). New formulation of the fourier modal method for crossed surface-relief gratings. J Opt Soc Am A.

[32] Rakić AD, Djurišić AB, Elazar JM, Majewski ML (1998). Optical properties of metallic films for vertical-cavity optoelectronic devices. Appl Opt.

[33] Malitson IH (1965). Interspecimen comparison of the refractive index of fused silica. J Opt Soc Am.

[34] Liu N, Guo H, Fu L, Kaiser S, Schweizer H, Giessen H (2007). Three-dimensional photonic metamaterials at optical frequencies. Nat Mater.

[35] Choi B, Iwanaga M, Miyazaki HT, Sakoda K, Sugimoto Y (2014). Photoluminescence-enhanced plasmonic substrates fabricated by nanoimprint lithography. J Micro Nanolithography MEMS MOEMS.

[36] Luo EZ, Heun S, Kennedy M, Wollschläger J, Henzler M (1994). Surface roughness and conductivity of thin Ag films. Phys Rev B.

[37] Lee HS, Awada C, Boutami S, Charra F, Douillard L, de Lamaestre RE (2012). Loss mechanisms of surface plasmon polaritons propagating on a smooth polycrystalline Cu surface. Opt Express.

[38] Hyuk Park J, Nagpal P, Oh SH, Norris DJ (2012). Improved dielectric functions in metallic films obtained via template stripping. Appl Phys Lett.

